# Thinking with and between social orders: Corporate embedded colleges and their public rivals

**DOI:** 10.1007/s10734-025-01415-1

**Published:** 2025-03-04

**Authors:** Morten Hansen

**Affiliations:** Department of Digital Humanities, King’s College London, London, UK

**Keywords:** Social order, Rival order, Higher education, International students

## Abstract

Scholars have argued that corporatisation is antithetical to higher education’s educational purposes. In this article, I complicate such claims by considering how higher education institutions develop concrete alternatives once a private coordination mode emerges as the dominant order. I also aim to show how we can use concrete practices and their content, purpose and structure to reveal alternatives to current market forms. By drawing on pertinent interviews and documents, I undertake a comparative analysis of the order of England’s corporate embedded colleges. Corporate embedded colleges are run by private companies in partnership with public universities. The purpose is to recruit and train international students for university preparatory programmes. I compare these colleges with three rival public orders: the University Pathways Alliance, in-house programmes, and the Northern Consortium. I proceed by thinking with and between these social orders. The goal is to help imagine alternatives to the corporate market for such colleges. I conclude that universities can learn from the private order and perhaps even outcompete its representatives if they cooperate and make full use of their institutional distinctiveness.

## Introduction

Embedded colleges — also known as corporate embedded colleges, international pathways, foundation colleges, or pathway colleges — are privately owned schools located on university campuses. These institutions deliver foundation programmes for international students (Komljenovic, 2016; McCartney & Metcalfe, 2018; Winkle, 2014). The industry’s genesis can be traced to a partnership signed in 1984 in Australia (Gillett, 2011). The English sector^1^ first started growing and gaining legitimacy between 2004 and the mid-2010s (Hansen, 2024). During this period, universities recognised that pathway partnerships could help them recruit more international students. Today, the market is dominated by five incumbents: Study Group, Into University Partnerships, Navitas, Kaplan International Pathways, and Cambridge Education Group. All five incumbents have revenues that exceed £50 million and have educated hundreds of thousands of students (Hansen, 2022). Such embedded colleges use market mechanisms (Aspers, 2009; Beckert, 2009) and the corporate form (Beckert, 2021; Chandler, 1992) to solve coordination problems for universities that relate to recruiting and educating foundation students.

There are, however, alternatives to corporate embedded colleges. In this article, I argue that we can open a dialogue between the market for corporate embedded colleges and public alternatives that solve similar coordination problems. Examples of the latter are in-house programmes, the University Pathways Alliance (UPA), and the Northern Consortium. In-house programmes are international pathway programmes run by universities themselves. The UPA is a network of universities with in-house programmes. The Northern Consortium is a charity governed by ten universities that specialises in accrediting its international foundation year programme, which is delivered by partner schools.

I selected these alternatives because they solve similar coordination problems to foundation programmes. That said, they have different ways of coordinating their activities and different reasons for doing so. Through my discussion of corporate embedded colleges and the alternatives, I seek to answer the following research question:

*Without seeking to directly change the broader institutional conditions they operate in, can universities reverse the outsourcing practices that potentially hollow out their educational purposes; and, if so, how?*

To this end, I introduce a theoretical vocabulary, one that allows me to discuss and compare (a) the markets in which corporate embedded colleges operate and (b) the higher education institutions that aim to solve similar problems. Inspired by Aspers (2010, 2011), I introduce the sociological concepts of ‘social order’ and ‘partial social order.’^2^ Note that I shall use these terms in very specific ways:

- A social order is an abstract concept that operates at the level of theory by making claims about how people act under specific conditions.
- A partial social order is the concrete instantiation of such an order, one that is captured through empirics and experience: it is what people do, how they do it, and why they do it.

I shall argue that the concept of partial social orders can help us see how seemingly antithetical ways of organising education-related activities are socially produced. These activities are, in turn, sometimes produced by the same actors and sometimes in complementary ways.

I then detail the four partial social orders studied in this article: the corporate order for embedded colleges, followed by the three rival orders. I reflect on the key practices in each order by drawing out connections between their content, purpose and structure. Doing so allows me to move beyond one-to-one comparisons between social orders. I can then critically reflect on how practices instantiate not only *what* English universities do but also *how* and *why* they do it. Focusing on issues of competition, cooperation and value, I next discuss the different ways in which these partial social orders solve similar coordination problems. It follows that although the orders solve similar coordination problems, understanding them in their context requires considering how and why they relate to larger structures and purposes at play.

Finally, I offer concrete suggestions for how universities can reverse the outsourcing of international foundation programmes: they can do so by combining some of the practices and structures deployed by the orders analysed in this article. I argue that the educational purposes of higher education (Ashwin, 2022) can, in turn, be protected through a plurality of evolving practices. These include practices that take inspiration from the way public universities and private corporations operate.

I intend to contribute to the higher education literature by building on both existing theoretical engagements with economic sociology (e.g. Komljenovic & Robertson, 2016; Lewis et al., 2022) and analytical efforts to unlock public–private dichotomies (Marginson, 2007; Szadkowski, 2019). The English universities I look at can be studied as both firms and public institutions because this is how they act and are being legislated. This is, however, not the same as assuming or stating that universities in general should be what some have described as public–private hybrids (Guzmán-Valenzuela, 2016). Instead, it involves opening a discussion about what universities can concretely do once their revenue sources, institutional forms and educational purposes have been outsourced, hybridised, or emptied out (Ashwin, 2020; Guzmán-Valenzuela, 2016; Marginson & Yang, 2023; McCowan, 2017; Newfield, 2016).

## Methodology

This article draws on a larger research project about corporate embedded colleges in England. I conducted 36 semi-structured interviews, spread across six groups of respondents: university leadership, university staff, corporate pathway staff, corporate executives, investors and representatives of the three public alternatives. Interviews were transcribed, anonymised and iteratively open-coded. Memos were written using NVivo (Kuckartz, 2014). This allowed for the identification of recurring themes and data reduction. It also aided continued rereading of, and immersion in, the transcripts (Miles & Huberman, 1994; Punch, 2014). Patterns within and across participant responses revealed how members viewed both their own and rival orders. Through this analysis, it emerged that the dominant order of corporate embedded colleges exists in a constitutive relationship with less-known rival orders.

As part of this project, I also collected documents relevant to the English corporate pathways market and its rivals. These documents included pathway and university annual reports, financial statements, strategy documents, quality oversight reports and secondary market analyses. I used these to immerse myself in the sector and understand how problems and solutions are framed in this space (see for example Bacchi, 2009 on ‘problem definition’). I also consulted the documents and my notes before and after conducting interviews. This allowed me to prepare for the interviews and reflect on the responses. The collected documents therefore played an important role in contextualising, prompting and elucidating my participants’ accounts of both the sector and their related experiences.

## Responding to analytical dichotomies through partial social orders

Higher education has a multitude of educational purposes (see Grubb & Lazerson, 2005; Zgaga, 2009; Kerr, 1990; McArthur, 2011). These include the development of skilled, reflective and critical individuals; the development of a critical, reflective, mobile and more equal society; and the development of religious values (Ashwin, 2022, p. 1233). Educational purposes are analytically distinct from higher education’s institutional form. That said, institutional forms directly affect the kind of development and learning opportunities universities offer (Ashwin, 2020), and these institutional forms are changing as the sector commodifies (McCowan, 2017; Robertson & Komljenovic, 2016). Higher education scholars have, consequently, remained sceptical about the private sector’s continued ‘creep’ (Ball & Youdell, 2008; Robertson & Dale, 2013; Verger et al., 2017). Indeed, a burgeoning literature has documented how higher education’s commercialisation can place pressure on such purposes as well as, by extension, on the very notion of a university (e.g., Collini, 2011, 2017; Jones et al., 2020; Newfield, 2016; Wright & Shore, 2017).

Bacevic (2019, p. 111–152) has identified entire genres of contemporary commentaries framing such processes as a ‘war on universities.’ Such war-like framings can lead to a ‘redemptive’ analytical commitment (Dale, 2001). Education institutions are a priori (i.e. independent of empirical study) seen as protectors of their educational purposes, while market-like ways of operating are seen as the opposite. In this view, market-like organisations must be eradicated from higher education to protect the latter’s educational purpose. In practical terms, this will, however, require major changes in contemporary capitalism, politics and associated notions of the knowledge economy (BIS, 2016; Harvey, 2005; OECD, 1996). Short of changing these broader conditions, what can universities do to fulfil their educational purposes as best as they can given the context in which they operate? If they have outsourced their teaching and recruitment to private companies, for example, then how could they reverse this process?

Sociological discussions of ‘order’ (Berger & Luckmann, 1967; Parsons, 1949; Weber, 1992) seem to provide a good point of departure for answering such questions. This is because they allow us to unpack and compare various social arrangements, the reasons for their continued existence and the conditions of their co-existence. In this article, I shall follow Aspers’ definition of order^3^ as ‘the predictability of human activities and the stability of social components in relation to each other’, which is ‘often seen as the antithesis of chaos’ (2010, p. 7). A *social* order is, therefore, an abstract concept, one that makes theoretical claims about how people act under specific conditions. Aspers’ approach to social orders is useful when studying higher education because he goes beyond thinking about orders as mere abstractions to presenting them as something that can and should be studied empirically. To do this, he introduces the concept of a ‘partial’ social order.

A *partial* social order is a concrete concept: it is what people do, how they do it and why they do it. Empirically speaking, existing orders are always partial because they are ‘a matter of degree’ (rather than a binary ‘is’ or ‘is not’). Existing orders also draw on the conventions, laws, institutions and social relations specific to other partial orders (Aspers, 2010, p. 7). Actors can, therefore, try to act ‘as if’ they are operating in perfect instantiations of some higher order. An example from Warren is an academic trying ‘to fabricate or perform a different self (that is more) conducive to measurement under the gaze of the (research excellence framework)’ (2017, p. 138). But actual practices will never be a carbon copy of any one idealised order. This is because, in our empirical reality, processes, meanings, relationships and identities are dynamic and changing (Berger & Luckmann, 1967; Vogel, 2018). Komljenovic and Robertson (2016) have, for example, shown how the making of markets in higher education are dynamic and sometimes transformational processes — processes where people’s roles and orientations can shift from situation to situation. In one instance, a university employee is a buyer of services; in another, the same individual is a seller. People can also orient themselves toward incompatible ideal orders at different times. In one instance, a scholar might critique the quantification of their research output impact; in the next, they might embrace the same logic to promote themselves and their work (Pardo-Guerra & Pablo, 2022).

The observation that all empirically existing orders are examples of a partial social order provides us with a novel way out of an analytical cul-de-sac, one that relates to viewing higher education in terms of a rigid dichotomy between public and private (Marginson, 2007; Szadkowski, 2019). Partial social orders thus provide a useful analytical vantage point for understanding practices in higher education systems as ‘traversed by a series of tensions and internal contradictions’ (Szadowski, 2017, p. 44). These contradictions, in turn, often emerge as loci for future institutional change (Baas et al., 2023; Brankovic & Cantwell, 2022; Clark, 1983). Leadership at a university can, for example, orient particular practices to norms and governance mechanisms that are internal to the university (Shattock & Horvath, 2019). At other times, the same leaders might put these norms aside. This could be done by outsourcing activities that are core to the institutions’ educational purpose, such as admission, teaching and research (Newfield, 2018). To further complicate matters, leaders can engage in these activities in the hope of generating new sources of income. These sources might — as Scott puts it — subsidise activities that are believed to reproduce the universities’ intellectual authority independent ‘from the political power on which this depends’ (1984, p. 26).

We can make normative arguments about what higher education should be like and should produce (Nussbaum, 2010; Pring, 2004). However, short of transforming the conditions within which it operates, we need an empirically grounded approach to consider how the university can get there. For example, if a university has outsourced teaching activities that are core to the institution’s educational purposes, then how can it reverse this process when the pressures that led to the outsourcing remain?

We know that the market order (an order in which competing public universities partner with competing private firms) has emerged as the dominant order. Nonetheless, I shall contend that we should examine existing alternative or ‘rival’ orders — those that have failed to achieve dominance. The common characteristic of alternative orders is that they stand in a rivalrous relationship to the dominant order. This means that they challenge the dominant order’s fundamental purposes and/or practices but operate in a similar space. As mentioned, the rival orders I study in this article are in-house programmes, the UPA, and the Northern Consortium. These are rivalrous public orders because they engage in practices that aid the recruitment and training of international foundation students.

Rival orders can interact, clash and build on each other. These mutually constitutive and competitive relationships might help explain why universities can act like public institutions in some instances and for-profit firms in others (Marginson & Considine, 2000; Zilwa, 2005). Orders come with their own assumptions and purposes, and institutions have some leeway to decide which orders they will draw from, when they will do so, and in which combinations. To unpack these activities, I propose that we think both *with* and *between* orders:

- Thinking *with* orders refers to evaluating, comparing and imagining orders relative to each one’s assumptions and purposes.
- Thinking *between* orders is the same exercise but grounded in a fusion of the orders’ assumptions and purposes.

By doing both, I intend to bring rival orders into dialogue with each other. For consistency and to draw out meaningful similarities and differences between the orders, I shall focus on key practices and relationships to concomitant *contents*, *purposes* and *structures*:

- By *content*, I mean the activities and practices that the interview respondents identified as being important. This might include teaching, recruiting and accreditation.
- *Purpose* refers to *why* practices are enacted in the first place. It underpins both a practice’s degree and type of legitimacy and unspoken norms and cognitive frames (Beckert, 2009). For example, extracting revenue from a charity for private gain counters the charity order’s purpose. Corporate firms doing so accords with their purpose and is therefore legitimate. What is and is not legitimate and what is or is not an order’s purpose is socially constructed and mediated by material and political realities.
- *Structure* expresses itself as a patterned reproduction of activities over time (Aspers, 2009). Structure is buttressed by legal forms of organisation and the ownership, institutions and norms through which actors exercise their agency. This might be the university leadership acting through a governance body, charity or private subsidiary.

**Fig. 1:**
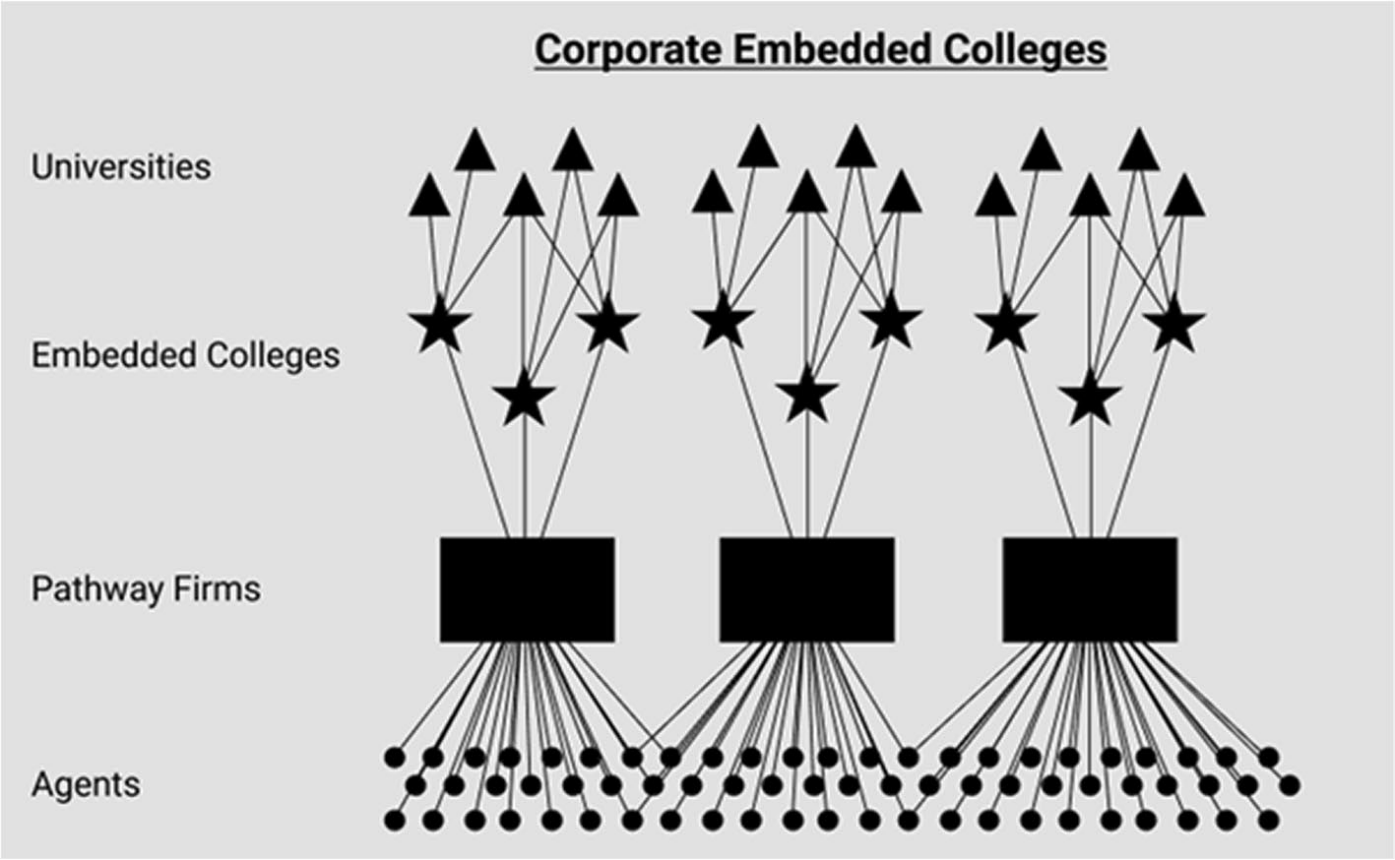
Corporate pathway firms — a corporate market order

**Fig. 2:**
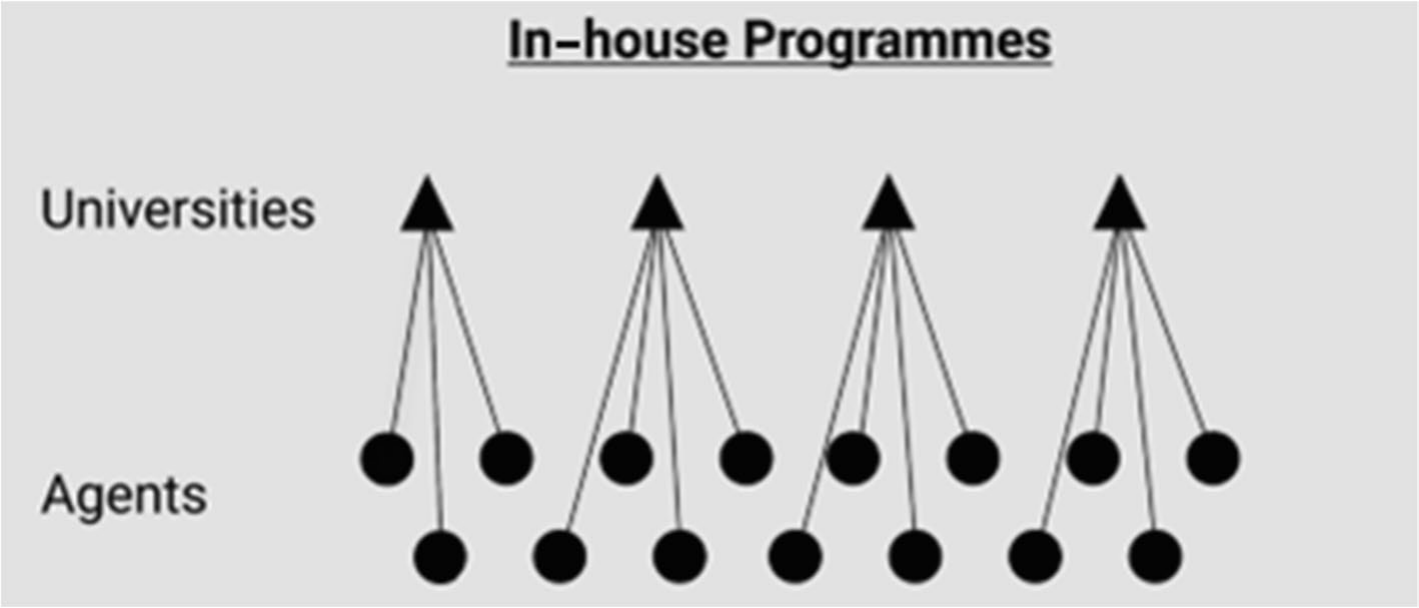
In-house programmes — a simple university order

**Fig. 3:**
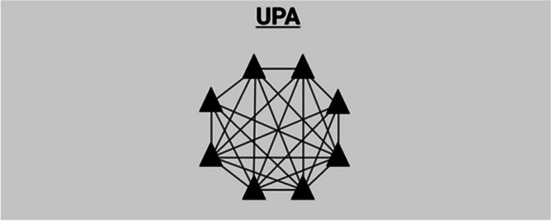
UPA — a cooperative network order

**Fig. 4:**
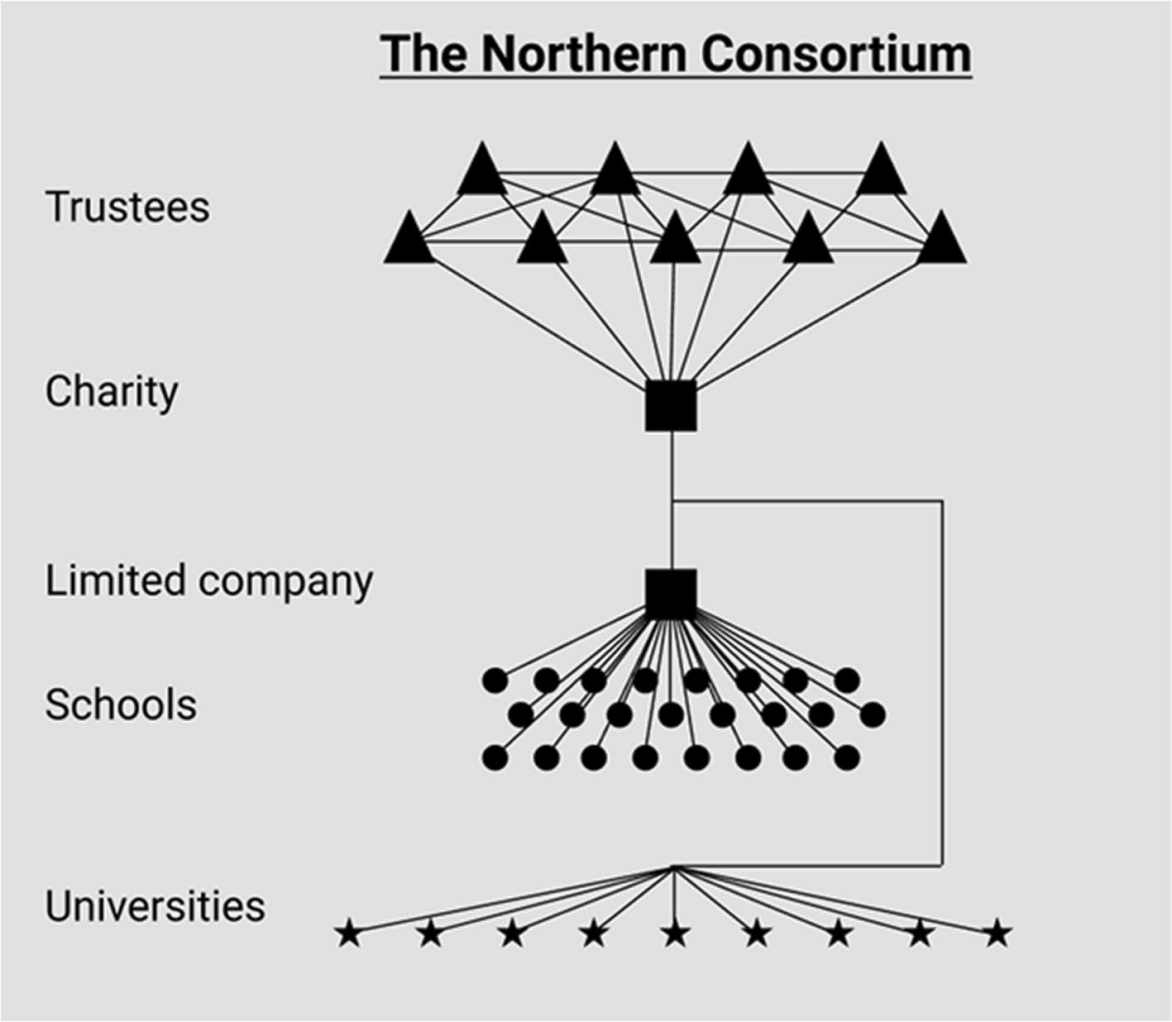
The Northern Consortium — a social enterprise market order

From this starting point, I can specify the historical relationship between the orders. This includes a chronology of which orders emerged first and how other orders were motivated by, built on or emerged as a reaction to previous orders. Inspired by Aspers’ schemas of markets and other modes of coordination (e.g. Aspers, 2010, pp. 8, 28, 85, 154), I have drawn a schematic structure for each order (Figs. 1–4). These schemas provide both the comparative foundation for my discussion and an overview of the ways in which an order might solve coordination problems relating to the recruitment and teaching of international foundation students.

I have outlined my view on rival orders and the relationships between practice and its content, purpose and structure. I now turn to the dominant corporate pathway order and then discuss the three relevant public alternatives.

## The dominating corporate order: pathway firms

Corporate embedded colleges have been described as a public–private partnership between universities and private firms (Komljenovic, 2016). The public university extends its brand to the embedded college, which is often located on campus. In return, the private provider recruits international students to the colleges, with the hope that they will eventually enrol at the partner university. Each corporate provider controls multiple embedded colleges and partners with a portfolio of universities and recruiting agents.

Corporate leaders and employees have claimed that a diversified portfolio of agents and universities allows them to promise ‘more and better students for less money’ (Executive, SE7). One executive member explained as follows:

The importance of the pathways to the agents is that they carry a portfolio of universities, and the ambition is that you have some which are very well-ranked and academically quite difficult to get into. And, you try and have a bottom-feeder or two, which is relatively easy to get into academically. The agent is then able to talk to its clients and say, look, I can get offers into these universities. Some of them are at the very top. If you are not good enough there, then you might get one in the middle and I’ve always got my insurance offer for you. Because most Chinese agents have moved to promising their clientele that they will get them five offers from UK universities, if they are looking at the UK only, then there is an arms race between the agents to offer their clients the best terms and the best options. And, what the pathways do is that they provide a portfolio that makes that easier. (Executive, SE8)

The study respondents further highlighted the economies of scale that these companies can offer universities, especially with regard to recruiting international students. This is a sector that continues to be reliant on advertising, managing a large number of in-country recruitment agents and strong application and visa processing capabilities. Corporate embedded colleges are seen as capable of doing this efficiently, partially because of how they structure their operations (for detailed analysis, see Hansen, 2022, 2024).

I have outlined this corporate order in Fig. 1. The bottom dots represent in-country recruiting agents. The lines from recruiting agents to corporate firms represent the flow of students from agents to firms and the flow of money from firms to agents. The students are then distributed to embedded colleges and eventually to partner universities. The relationships between embedded colleges and universities are rather competitive. This means that students can apply to any of the partner universities, although these universities hope that the ‘thick’ social bonds students have established on campus will make them more likely to stay at that institution. The actual conversion of pathway students to university students is more complicated. One Pro-Vice-Chancellor explained how they lost some of their best pathway students to a nearby Russell Group university:

The danger is that students who do particularly well on the pathway course can choose to go anywhere. Some of them do so well that they’ll get access into, you know, [a Russell Group University], which is tricky for us. (Pro-Vice-Chancellor, number removed to protect anonymity)

Universities are also subject to the general rules set by the corporate firm. The potential amount of follow-on commission paid to the recruiting agent is, for example, administered by the firm. Direct and open competition thus takes place both among corporate firms (over students, which materialises through a battle over recruiting agents and university relationships) and among universities (who generally want to increase their student numbers).

## The ‘traditional’ university order: in-house provisions

The in-house provision of foundation programmes is the order that most resembles the teaching arrangement we have come to expect from universities. I call this the ‘traditional’^4^ university order, where the public university owns and delivers the international foundation year. Goldsmiths, University of London, for example, offers an International Foundation Certificate. ‘[O]n successful completion’, this certificate will ‘guarantee a place on a relevant Goldsmiths degree programme’ (Goldsmiths, 2021). The foundation certificates are run by the Centre for Academic Language and Literacies, whose status is similar to that of a university faculty. A dozen or more universities have in-house provisions, including the University of Kent, the University of Warwick, the University of Birmingham, and Oxford Brookes University.

Figure 2 contains a schema of the simple university order, where individual universities are at the centre of coordinating student flows into their own in-house programmes. The triangles represent the universities hosting in-house foundation programmes, and the dots are recruiting agents. The connecting lines between the agents and the university represent the flow of students from recruiting agents to universities and the flow of commission payments from universities to recruiting agents. One university can have commercial relationships with well over 100 different agents.

The figure itself is more static and less complex than the actual social structures it points to. For example, not all students will continue to study at their foundation university. Some will travel back to their home country while others will continue studying at a different English university. The point is that this is not the structure’s goal or purpose. The structure is geared toward feeding a single pathway programme, one that prepares students for a single university in a way that keeps a manageable number of institutions involved in the process. This is both a strength and a weakness. On the one hand, it provides the university with a great degree of control over its teaching quality and strategy. On the other hand, it makes the in-house programmes vulnerable to private providers who promise to scale student recruitment. One university staff member described the dilemma as follows:

Even if you do [in-house pathway programmes] really well, even if you’re providing outcomes that are absolutely top-notch, the strength and capacity capability of the private sector to deliver numbers is very difficult to compete with. (University staff, SE18)

In this order, the university is seen as a legitimate actor in its own right, and the in-house provision is an extension of it. Legally, in-house pathways tend to be full members of the university rather than stand-alone subsidiaries, and their structures and purposes align with the university’s own. It is noteworthy that the corporate providers have aimed to copy this model of legitimacy as a marketable service. They do so by using university branding in their marketing materials and housing their teaching facilities on or near university campuses.

## The cooperative networking order: the UPA

The UPA provides a platform for networking and sharing information between in-house providers. The magazine *InForm* (first published in 2008) and the annual InForm conference (first held in 2010) can be considered precursors to the UPA*.* Most of the *InForm* magazine consists of articles written by academics about didactic, pedagogical and educational challenges and innovations. However, its leadership exercises an inclusive ethos by also welcoming contributions from people working in the corporate order. A person familiar with the journal explained that.

there was no way to – and in a way, there was no point in trying to – constrain the readership of the contributors to that journal to people who were in-house providers. Indeed, more and more over time, we could see that the in-house providers were falling to the private providers. Our good colleagues, who are still good colleagues, and were now working for a private provider, still had good things to say about pedagogy in the classroom and influential factors associated with the pathway. So, it wasn’t right to exclude them either. (Number removed to protect anonymity)

A sense of solidarity with the ‘good colleagues’ who had transitioned to the private sector is possible because the magazine’s purpose was not profit maximisation. It was also not conceived as a way to equip in-house programmes with a competitive advantage over corporate embedded colleges. The InForm conference similarly accepts submissions from professionals working at corporate embedded colleges.

Still, in-house providers needed a more involved way to exchange expertise. In 2014, *InForm*’s first editor and first Chair of the Editorial Board — Dr Anthony Manning — set up the UPA together with a handful of other member universities. The goal was to create a new forum for networking and exchanging best practices. The main mode of knowledge-sharing at the UPA occurs between its members and is ‘currently instigated by invitation only’ (UPA, 2020). In this way, UPA extends *InForm*’s cooperative structure, although it does make the network more exclusive by erecting membership barriers. Knowledge-sharing now happens at three to four annual meetings between the steering group members and at the yearly conference.

As with InForm, sharing good educational practice is still the UPA’s main purpose. That said, the latter displays greater awareness of the commercial landscape. This is evident in the way it projects itself and its members. Their claim to educational excellence is projected through the legacy, experience and prestige of members. The UPA was, however, not seen as standing in direct opposition to the corporate order. This is partly because there was still an emphasis on developing a space for education professionals to exchange pedagogical knowledge rather than only education exporters exchanging market information about how to attract potential students. One UPA member explained as follows:

So, the aim of it has never been really to challenge what private providers can do because we are not. The alliance [UPA] is not a group of people providing the same thing, we are each providing our own programme as we always were, but the UPA has a mechanism to share good practice in terms of core structure, academic innovation, [and] student support mechanisms. We share intel on recruitment patterns and marketing initiatives, but we don’t all recruit in the same way or at the same level. This is because we’re all working in different institutions, and it isn’t an effective, joined-up marketing opportunity. (Number removed to protect anonymity)

Figure 3 shows the UPA’s structure, which is depicted as a network of universities. Each triangle represents a member university and each line between them represents a social bond. Although the UPA has a steering group, a chair and membership fees, it is not a separate legal entity. Its cohesion depends on voluntary engagement because members value sharing their insights and experiences. The order also does not generate any significant cash, and it is not its purpose to do so.

## The social enterprise market order: the Northern Consortium

The Northern Consortium is a charity accrediting international foundation programmes and was launched in Manchester. In 1987, the University of Manchester Institute of Science and Technology (UMIST) struck a deal with the Malaysian government to provide a large number of students with access to English universities, particularly in the field of engineering. Many of these students were identified as needing additional training. Because the potential number of Malaysian students was higher than UMIST could handle on its own, the university set out a collaborative framework for foundation programmes, one that could be delivered in Malaysia (NCUK, 2018).

The group was originally organised as an administrative unit within UMIST and drew on the membership of 12 institutions.^5^ The mix of institutions contrasts with most universities’ aversion to being associated with institutions perceived as less prestigious based on positions in national university rankings. This mix was a recurrent theme in my interviews with both corporate executives and university leaders. It demonstrates a different sense of community, where rank does not dictate the ability to cooperate:

There’s no way UMIST could cope with that quantity. So, in a bit of panic on a Friday afternoon in October 1987, the dean of the faculty got on the phone and invited as many northern universities as he could. And remember, it was much more of a democracy then. There was not this great perceived competitive field. But [today] there’s an enormous amount of snobbery in this sector as well. (Senior leader familiar with the Northen Consortium, SE32)

By 1993, the organisation had formalised into a registered charity limited by guarantee. This means that it must act in a way that accords with its charitable objectives (see Companies House, 2019, p. 24). In 2003, the consortium founded a private subsidiary — now called the Northern Consortium UK Ltd. (NCUK). The subsidiary is the main vehicle through which the charity’s day-to-day business is implemented. Today, its main function involves accrediting and quality-assuring the NCUK curriculum taught at over 100 local study centres worldwide. An NCUK qualification is recognised by NCUK university members, and the NCUK helps students gain qualifications to enter its universities.

Figure 4 displays the structure of the Northern Consortium. The dots illustrate partner schools that deliver the NCUK curriculum. These are connected to the private arm of the consortium known as NCUK. The lines between them represent both oversight services from the NCUK to the partner schools and money flowing from the schools to the company in the form of fees and reputational capital. Below sits the charity to which the company answers. Ten trustees sit in the charity, all of whom have full-time jobs at the founding universities. Below that sit all the universities that accept NCUK credentials, which includes founding members and associated universities. The universities receive students from the limited company.

I have reviewed the structure, purpose and content of the dominant order and its three rivals (summarised in Table 1). I now examine the common need they are addressing. This is the coordination of activities surrounding teaching and recruiting international foundation students.

**Table 1:** Orders and their practices’ content, structure and purpose

	Content (‘What’ is provided?)	Structure (‘How’ is it provided?)	Purpose (‘Why’ is it provided?)
Corporate Embedded College	International foundation programmes and recruitment by leveraging scale of operations across multiple university partners and recruiting agents	Multinational corporation, often funded by private equity firms	To accumulate wealth for its owners
In-House Provision	International foundation programmes run by the university	Division within the university	To teach and funnel international students
The UPA	Regular meetings and conferences between in-house providers	Network of universities	To cooperate across universities by sharing teaching and recruitment experiences
The Northern Consortium	The NCUK curriculum, quality assurance and charitable activities, such as scholarships	Charity with a private subsidiary	To increase access to higher education

## Discussion

Corporate embedded colleges have been criticised by the University and College Union (UCU, 2008, 2012, 2014), academics (Komljenovic, 2016; McCartney & Metcalfe, 2018; Tamtik, 2022; Winkle, 2014) and journalists (The Guardian, 2011; Times Higher Education, 2014; The Times, 2024). Spurred on by broader institutional and structural changes (BIS, 2016; Bolton, 2021; House of Lords, 2023; OECD, 1996), these colleges have, nonetheless, managed to grow rapidly. As mentioned, this is largely because universities partner with them to recruit more international students and solve a coordination problem between agents and universities. Interview respondents repeatedly indicated that the corporate order’s popularity hinges on the ability to recruit international students by scaling operations across a portfolio of partner universities and recruiting agents. The rival orders that sprang up to address similar coordination problems (albeit in different ways) failed to become dominant orders. Following Beckert (2009), I now discuss the various orders’ relative merits by unpacking their ability to solve pertinent competition, cooperation and value problems.

### Competition problems and growth

Corporate embedded colleges structure competition over international students by competing directly with each other on behalf of their partner universities (Fig. 1). Part of the revenues that embedded colleges earn by cooperating with their partner universities is used to develop centralised and corporate recruiting capabilities, branding campaigns and market intelligence-gathering mechanisms (Hansen, 2022). This means that by sharing their resources with the same private partner, public universities also share resources with each other while competing for the same students. The irony of this order is that it partially emerged because universities initially resisted working directly with recruiting agents and generally did not want to cooperate with competing universities (Hansen, 2024). One university staff member put it this way: ‘Frankly, a lot of it is about whether or not you are being competitive with other UK institutions. It’s about growing your market share’ (University staff, SE17). Taken together, this hindered universities from developing a more self-reliant order for student recruitment and training.

The Northern Consortium is the closest example of universities coordinating student flows among themselves by funnelling students from accredited schools to member universities. The organisation does not see itself as a significant competitor to the large corporate pathways. Nonetheless, one corporate executive explained how Study Group’s first university agreements were inspired by the precedence that the Northern Consortium set. As they explained:

There was already a model for [establishing articulation agreements with UK universities]. There was something called the Northern Consortium of British Universities that was already doing that primarily in Malaysia. But that grew to become a majority of [Study Group’s] business. (Private Executive, SE31)

In other words, there was a time when the cooperative order was sufficiently innovative to warrant imitation by the emerging private order. Over the next 30 years, Study Group grew into a large multinational operation, one that stands in contrast to the Northern Consortium’s relatively modest operation and revenue.

The difference in growth has to do with the fact that they deliver different services: one accredits education programmes, while the other delivers education services directly (which involves a more capital-intensive operation). The other factor is that Study Group pursued an aggressive growth strategy, while the trustees of the Northern Consortium did not. This should not be surprising given each order’s different purposes (Table 1). Nonetheless, according to the study respondents, the slower growth was not only a product of the Consortium’s charitable objective. In fact, respondents claimed that the leaders actively used the charity’s objective to push university employees sitting on the board (i.e. the trustees) towards adopting a more growth-oriented posture (by, e.g. increasing the number of partner universities).^6^ One respondent familiar with this discussion explained as follows:

[The] view [of member universities] was, ‘well, if you bring more recipient universities into the pool, we may get a smaller [student] share’, so they weren’t that enamoured of that prospect [increasing the number of recipient universities] … It was clear that there was a huge scope and we had to convince both the charity trustees and the broader recipient pool of universities that growth was going to be in their interests or could be in their interests … [We had to] convince the charity trustees as a specific group that their obligations as charity trustees were not to protect the interests of the universities that they came from, but that, under charity law, their obligation was to act purely in the best interests of the charity, and the core objective of the charity was the advancement of education. (Senior respondent, SE34)

This use of charity law exemplifies how a social enterprise’s purpose and structure can be used to compel universities into taking a more proactive role in growing a charity’s operations. It also shows how the corporate order’s dominance partly spreads through constructing subjectivities, to the point that even university representatives working as charitable trustees can feel obliged to increase their institutions’ revenue.

It is fair to speculate about whether the Northern Consortium could have grown into a co-equal and semi-public alternative to the corporate order given its first-mover advantage and unique access to esteemed UK universities. This might have occurred if universities had continued to build more boldly on the foresight UMIST showed when establishing the charity. It is impossible to establish why this did not happen. That said, respondents generally indicated that universities were resistant to cooperating around international recruitment and there was a limited appetite to develop competitive public alternatives to the private recruitment sector. For example, when I pressed a respondent to explain why the consortium had not engaged in more expansive and capital-intensive collaborations around teaching and recruitment, they answered as follows:

You’re asking what appear to be competitors to come together … It took a huge amount of my energy to hold [the 10 or 11 members] together. Now, maybe I got it wrong in the way that I did it, but I had to work extremely hard at that stakeholder group there to retain them, to engage them, and to help them to take a deep interest in the strategy of the charity and to see this as being a valuable vehicle. Academics don’t get motivated by those sorts of things. (Number removed to protect anonymity)

This suggests that the corporate order has an advantage over the public orders because of its actors’ proactivity. One senior executive described it to me like this: ‘[There was this] mantra: “Invest ahead of the curve”; so, plan for success. We had too big a management team for the size of the business. But we grew into it by then [showing me on a graph]. We started to hire ahead-of-the-curve salespeople’ (Senior Executive, SE7). This mantra allowed the corporate order to develop the capacities to stand ready with clear solutions when public universities looked to increase their student numbers. This meant that trust developed between the respective public and private actors over time.

### Cooperation problems and trust

Cooperation problems often require actors to orient themselves toward a shared imagined future (Beckert, 2016). Partner universities’ trust in the corporate orders’ ability to deliver on the future they were promising played an important role in this regard. At the highest institutional level, trust between university and corporate leadership was built by delivering on promises to increase university revenue and through repeated and close cooperation. One corporate executive explained as follows:

Our aspiration is to say that the heart of what we are is a good partner to universities. They trust us. So, unlike a lot of university outsource arrangements – and let’s be honest, it’s a sort of outsourcing arrangement – I will have a relationship with the vice-chancellors of our partners. You know, if we were doing frankly anything else in the business, I wouldn’t have that kind of relationship with the university. But international students are the strategic part of what they do and, for some of our core partners, we bring in a lot of revenue. And, that then puts us in a really good position to think about the other services that we can add of value. (Private Executive, SE22)

In operational terms, trust allows the progressive unlocking of economies at scale across university portfolios. This is because universities trust corporate firms to strike a balance between growing their own and the individual universities’ revenue. The result is that private firms are strategic about how they develop personal trust with specific university staff members (Komljenovic, 2018).

One pathway staff member explained how trust between university and private actors can emerge over time as they both climb their respective organisations’ hierarchies:

With Kaplan, like INTO, you have people who had been there for 10 to 12 years. You see the same through universities. And, because of that you can build trust because they are normally in the same position as well, and they move up. So, you have really strong relationships – really strong connections going back 10, 15, 20 years, however long it may be. (Corporate pathway staff, SE2)

In other words, trust is fostered both by projecting a common future (of realised revenues) and by sharing a common future (through sustained personal relationships).

The connection between trust and revenue represents a relative weakness for rival orders operating in a context where universities are increasingly expected to grow their own sources of revenue. While the UPA, for example, is founded and operates on notions of trust and solidarity, the formal content and structure of its practices do not allow for substantial revenue-generation activities — activities that can be scaled through re-investments into the UPA structure itself. It does not use this trust to coordinate significant and sustained structural market advantages. A similar dynamic was at play in the Northern Consortium, where the NCUK originally had to transfer its profits to the charity (Fig. 4). This complicated the re-investment of operational profits into growth-oriented activities (SE32–34, 36). That said, both orders provide a structure for trust to develop between member universities, which is all but precluded in the corporate alternative.

### Value problems and pricing

Markets solve value problems through pricing. The commission prices that corporate and in-house providers are willing to pay to recruiting agents depends on what they consider appropriate and necessary given their organisation’s purpose, the category of the good they are selling and the brand they command. In the corporate order, all valuations of commission, foundation and accommodation fees are negotiated through the pathway firm. The same applies to the subsequent revenue distribution. Because the firms’ purpose is to maximise profit, they are not bound by the same normative and legal standards as in-house provisions. One pathway executive made this point as follows:

We pay commercial terms and rates that universities would never dream of paying and we are a lot more agile and nimble in the way we work because we are not as restricted. Universities are essentially governed organisations and they have charitable status. (Pathway executive, SE27)

The in-house providers I spoke to tended to see their programmes as a class of goods or services distinct from corporate providers. This is because they tended to be full members of their universities. Their pricing practices and decision-making processes also aligned more with those of traditional universities than corporate providers. This means that in-house providers were less dynamic, less aggressive, less centralised and more driven by governance structures endogenous to their respective universities. A person knowledgeable about the UPA and responsible for an in-house provision explained the difference:

**Respondent**: The [corporate pathways] are commercial, so they can incentivise their agents differently. We [universities] are quasi-government. So, we cannot incentivise agents other than being grateful. We cannot give them things – iPads and so on. So, they can incentivise students in a way that [we] cannot.

**Interviewer**: So, you can pay commission like the rest of the market, but you can’t give gifts and host extravagant dinners and so on?

**Respondent**: Yes, partly we cannot and partly that is not the way we work. (Member of staff at in-house provider, SE37)

Most corporate pathways are partly owned by private investors who can generate value in two ways: through tuition fees and by selling their pathway business. This can incentivise corporate pathways to overpay recruiting agents (or ply them with gifts) to boost growth numbers, which raises the student acquisition cost for the entire sector (Hansen, 2024).

A less aggressive pricing approach might be seen as one of the public order’s weaknesses because it can reduce the ability to grow rapidly. Nonetheless, for universities, steady organic growth in student numbers might improve both leadership’s ability to manage its financial exposure and academics’ abilities to design and deliver research-led courses. Universities are sometimes ‘slow’ because those practices align with their underlying educational purposes. From this vantage point, steadiness is a strength rather than a weakness. This is because the potential inertia of in-house provisions reflects the virtues of universities as democratic institutions and keepers of knowledge — virtues that steadily develop in line with academic priorities. Decisions are taken by the council and senate following dialogues with the faculties. It has proven difficult to extend such decision-making processes outside the university and into endeavours such as capital-intensive cooperations between a trusted group of universities.

## Conclusion

Examining partial social orders deepens existing research. As Robertson and Olds suggest, education markets are ‘instituted, or produced, through social institutions, and legal and political strategies’ (2012, p. 10; see also Muellerleile & Lewis, 2019; Lewis et al., 2022). Such scholarship has shown that universities can simultaneously operate like private firms and public institutions (Marginson & Considine, 2000; Muellerleile & Lewis, 2019; Shattock & Horvath, 2019). This denies a straightforward dichotomy between private orders and public purposes. As the empirical reality of the market for corporate embedded colleges demonstrates, universities are faced with complex trade-offs — trade-offs that are both normative and pecuniary in nature. Private providers exploit these complexities by standing ready with outwardly clear solutions.

Thinking with and between partial social orders provides a systematic framework for analysing such choices. To denaturalise the now dominant corporate order, I set out to examine the rival orders that emerged to address similar needs. In doing so, I articulated these orders’ strengths and shortcomings. I also discussed potential courses of action for universities seeking to reverse outsourcing practices without directly changing the broader conditions they operate in.

Looking at the rival orders helps us substantiate how the corporate order’s success is not grounded in characteristics that are unique to the order per se. They are, instead, a consequence of how and why coordination problems between agents and universities are solved. For example, the efficacy popularly credited to corporate firms sprang from how they work together with multiple partner universities to achieve scale rather than from their for-profit purpose (Hansen, 2024). Yet, as the Northern Consortium case demonstrated, universities could do this without private involvement. They choose not to because they see each other as competitors. As a result, universities end up de facto cooperating through the embedded colleges — each becoming assets in a private company’s portfolio of universities. By opting for cooperation through an intermediary (i.e. a multinational corporation) over direct cooperation with each other, universities limit their influence on how the sector is shaped. In the process, they hand over revenue and market power to the private sector and its investors.

Notable advantages that the corporate order has over rival orders are speed and an obsession with growth. Most of the embedded colleges are owned by private equity, which means that the owner(s) can accrue wealth through operational profits and by selling the business. The faster the company grows, the more valuable it becomes in global financial markets. This means that the value of growing student numbers — even when overpaying recruitment agents — might have a multiplier effect on the value that can be realised when selling the company. This is something that a public university cannot copy via rival orders because scaling education for (potentially speculative) financial gain stands in tension with a university’s educational purpose (Nussbaum, 2010). Nonetheless, it is important to emphasise that this is de facto a business model that universities are supporting when signing up with private equity-owned providers, even though the universities cannot ostensibly access any of the potential windfalls.

What, then, is the rival orders’ value? The UPA and the Northern Consortium are both explicitly cooperative in their purpose. They challenge the view that universities must compete with each other if they are going to thrive. Together with in-house providers, these rival orders focus on delivering education over sales. Universities might increase student numbers without involving corporate embedded colleges if they combine these rival orders’ practices and structures. In-house providers could, for example, use the UPA as a formal structure for centralising cooperative and resource-intensive recruitment and branding efforts. To manage risk and shield universities from the risks of bankruptcy-related losses will require the establishment of a private arm with the legal protections of a limited liability firm (as seen with the Northern Consortium and the NCUK). Another option is that universities currently partnering with embedded colleges might also partner with other universities to run a joint embedded college, thereby displacing the private partner. Freed from private investors who push to extract value and accelerate growth (Hansen, 2024), the key to a successful venture could involve (a) splitting overheads on teaching and recruitment costs and (b) recruiting a diverse student body that can be placed at a diverse set of partner universities.

The rival orders also have unique educational strengths. Part of universities’ value lies in determining what knowledge is taught, who teaches it and under which conditions. It may, for example, be a good trade-off to have smaller foundation programmes while remaining in control of teaching and student bodies (even if overall profits are lower than in the corporate order). In this context, the rival orders’ ‘slow’ speed is a strength, one that preserves educational purpose and associated democratic modes of governance. Universities embedded in these rival orders also value the long-term development and protection of knowledge, which privileges stability over volatile growth. This is particularly important when considering private markets’ tendency to follow boom and bust cycles. Steady organic and cooperative growth can insulate universities from these cycles over the long run, thereby accentuating the public distinctiveness of these important institutions.

## Data Availability

The data from this study is not made publicly available to preserve the anonymity of research participants and their place of work.

## Footnotes

1 This study is part of a larger research project, which focused on corporate embedded colleges in England. I did not interview respondents working for universities in the other UK countries, Wales, Scotland, and Northern Ireland.

2 To improve readability, I do not always use the qualifiers ‘social’ and ‘partial,’ when speaking about partial social orders. This follows the convention set by Aspers.

3 Like Aspers (2011), I make use of the qualifier ‘social,’when speaking about order to connote that the orders in which I am interested are expressed by, and thus partially produced through, social interactions, relations, conventions, laws, institutions, expectations, and meaning-making processes (Beckert, 2009; Granovetter, 1985; Polanyi, 2018; Weber, 1992).

4 For Scott, the traditional university is associated to two ideal types: the ‘Liberal University’ and the ‘Modern University’ (Scott, 1984, p. 26).

5 This number was reduced over time to ten because UMIST and Victoria University merged into the University of Manchester, and the University of Liverpool exited the charity in 2016. As a part of the 1992 reform, five of the polytechnic members changed status to become universities.

6 It is outside the scope of this article to discuss all the reasons for why the Consortium did not grow faster (Hansen, 2022).
